# Lipid mediator n‐3 docosapentaenoic acid‐derived protectin D1 enhances synaptic inhibition of hippocampal principal neurons by interaction with a G‐protein‐coupled receptor

**DOI:** 10.1096/fj.202101815R

**Published:** 2022-02-21

**Authors:** Apostolos Mikroulis, Marco Ledri, Gabriele Ruffolo, Eleonora Palma, Günther Sperk, Jesmond Dalli, Annamaria Vezzani, Merab Kokaia

**Affiliations:** ^1^ Epilepsy Center Department of Clinical Sciences Faculty of Medicine Lund University Lund Sweden; ^2^ Department of Physiology and Pharmacology Istituto Pasteur‐Fondazione Cenci Bolognetti University of Rome Sapienza Rome Italy; ^3^ IRCCS San Raffaele Pisana Rome Italy; ^4^ Department of Pharmacology Medical University Innsbruck Innsbruck Austria; ^5^ William Harvey Research Institute Barts and The London School of Medicine and Dentistry Queen Mary University of London London UK; ^6^ Centre for Inflammation and Therapeutic Innovation Queen Mary University of London London UK; ^7^ Mario Negri Institute for Pharmacological Research IRCCS Milan Italy

**Keywords:** antiepileptic mechanism, GABA_A_ receptors, mouse hippocampus, PD1_n‐3DPA_, perisomatic inhibition

## Abstract

Epilepsy is a severe neurological disease manifested by spontaneous recurrent seizures due to abnormal hyper‐synchronization of neuronal activity. Epilepsy affects about 1% of the population and up to 40% of patients experience seizures that are resistant to currently available drugs, thus highlighting an urgent need for novel treatments. In this regard, anti‐inflammatory drugs emerged as potential therapeutic candidates. In particular, specific molecules apt to resolve the neuroinflammatory response occurring in acquired epilepsies have been proven to counteract seizures in experimental models, and humans. One candidate investigational molecule has been recently identified as the lipid mediator n‐3 docosapentaenoic acid‐derived protectin D1 (PD1_n‐3DPA_) which significantly reduced seizures, cell loss, and cognitive deficit in a mouse model of acquired epilepsy. However, the mechanisms that mediate the PD1_n‐3DPA_ effect remain elusive. We here addressed whether PD1_n‐3DPA_ has direct effects on neuronal activity independent of its anti‐inflammatory action. We incubated, therefore, hippocampal slices with PD1_n‐3DPA_ and investigated its effect on excitatory and inhibitory synaptic inputs to the CA1 pyramidal neurons. We demonstrate that inhibitory drive onto the perisomatic region of the pyramidal neurons is increased by PD1_n‐3DPA_, and this effect is mediated by pertussis toxin‐sensitive G‐protein coupled receptors. Our data indicate that PD1_n‐3DPA_ acts directly on inhibitory transmission, most likely at the presynaptic site of inhibitory synapses as also supported by *Xenopus* oocytes and immunohistochemical experiments. Thus, in addition to its anti‐inflammatory effects, PD1_n‐3DPA_ anti‐seizure and neuroprotective effects may be mediated by its direct action on neuronal excitability by modulating their synaptic inputs.

**Abbreviations**
aCSF: artificial cerebrospinal fluid
AMPA: α‐amino‐3‐hydroxy‐5‐methyl‐4‐isoxazolepropionic acid
AP‐5: (2R)‐2‐Amino‐5‐phosphonopentanoic acid
ASDs: antiseizure drugs
BBB: blood‐brain barrier
CA: cornus ammonis
CTZ: cyclothiazide
DHA: docosahexaenoic acid
EPA: eicosapentaenoic acid
GABA: gamma aminobutyric acid
GPCR: G‐protein coupled receptor
HRP: horseradish peroxidase
IL‐1β: interleukin 1β
NBQX: 6‐nitro‐2,3‐dioxo‐1,2,3,4‐tetrahydrobenzo[f]quinoxaline‐7‐sulfonamide
OR: oocyte ringer
PD1_n‐3DPA_
: n‐3 docosapentaenoic acid‐derived protectin D1
PFA: paraformaldehyde
PTX: pertussis toxin
PUFAs: poly unsaturated fatty acids
ROD: relative optic densities
s(m)I(E)PSCs: spontaneous (miniature) inhibitory (excitatory) post‐synaptic currents
TLE: temporal lobe epilepsy
TNF: tumor necrosis factor

## 1 INTRODUCTION

Epilepsy is one of the most common and devastating neurological disorders affecting about 1% of the world population. Available antiseizure drugs (ASDs) are mainly providing symptomatic relief and fail to adequately control seizures in about 40% of patients. Alternative treatments include surgical removal of the seizure focus, vagal nerve, and deep brain stimulations, as well as dietary supplementation such as ketogenic or PUFAs‐enriched diet.^1^ However, these treatments offer variable efficacy outcomes and surgery can be proposed only to a restricted number of patients.^2^ Therefore, there is a high demand to develop novel treatments for epilepsy based on a better knowledge of the pathogenic mechanisms underlying seizures.

Neuroinflammation has been described as a common pathogenic mechanism promoting seizures in animal models of acquired epilepsy, and common forms of human drug‐resistant epilepsy.^3^ Pharmacological interventions with specific anti‐inflammatory drugs provided evidence in support of a pivotal involvement of cytokines, chemokines, complement factors, and prostaglandins, in seizure generation and recurrence.^4^ Notably, inflammatory molecules contribute to increasing neuronal network excitability by various mechanisms, which include modulation of synaptic transmission,^5^, ^6^ changes in blood‐brain barrier (BBB) permeability, consequent excitatory synaptogenesis,^7^ and alterations in glial cell function.^8^ Altogether, these changes lead to pathophysiological de‐regulation of neuronal networks promoting hyper‐synchronization and seizures.^3^, ^5^, ^9^ It has been proposed that neuroinflammation in epilepsy persists due to inefficient engagement of resolving mechanisms, as supported by evidence that anti‐inflammatory agents decrease seizures by reducing neuroinflammation.^3^

Endogenous pro‐resolving systems operate both in peripheral tissue and CNS and include various proteins and key lipid mediators derived from n‐3 PUFAs such as lipoxins, resolvins, protectins, and maresins.^10^ One such resolving molecule has been recently identified by us as n‐3 docosapentaenoic acid‐derived protectin D1 (PD1_n‐3DPA_), which increases to an insufficient extent in the hippocampus of mice developing epilepsy.^6^ In fact, supplementation of PD1_n‐3DPA_ during epilepsy development by its intracerebroventricular injection, reduced recurrent spontaneous seizures as well as mRNA levels of IL‐1β and TNF ictogenic cytokines. Comorbidities, such as weight loss and impaired cognitive function were also ameliorated. Although the resolution of neuroinflammation by PD1_n‐3DPA_ is likely involved in its therapeutic effects, this lipid mediator might also affect network excitability and synaptic transmission by yet unknown direct mechanisms. In support, there is evidence that n‐3 PUFAs (such as EPA, DHA) decrease evoked hippocampal neuronal excitability in brain slices.^11^, ^12^

To address this hypothesis, we incubated naïve mouse hippocampal slices with PD1_n‐3DPA_ and investigated its effect on excitatory and inhibitory synaptic drive onto the pyramidal neurons of the CA1 region. We demonstrate that PD1_n‐3DPA_ increases inhibitory synaptic drive onto the pyramidal neurons mediated by GPCRs and thereby may counteract the increased excitability of hippocampal network resulting in seizure inhibition.

## 2 MATERIALS AND METHODS

### 2.1 Animals

Mice with C57BL/6 background were bred and housed (5 mice/cage) in the SPF facility at a constant room temperature (23°C) and relative humidity (60 ± 5%) with free access to food and water, and with a fixed 12 h light/dark cycle. All experiments were approved and conducted according to the Lund/Malmö experimental animal committee ethical permits 02998/2020 and M47‐15.

### 2.2 PD1_n‐3DPA_ solution preparation

For electrophysiology in slices or *Xenopus* oocytes, as well as for GABA_A_R immunohistochemistry, the PD1_n‐3DPA_ (10 mM in ethanol) was kept at −80° until the experiment was started. Immediately before the incubation, the ethanol was evaporated with nitrogen gas and the compound was solubilized in 450 µl artificial cerebrospinal fluid (aCSF) for experiments in slices (118 mM NaCl, 2 mM MgCl_2_, 2 mM CaCl_2_, 2.5 mM KCl, 26 mM NaHCO_3_, 1.25 mM NaH_2_PO_4_, and 10 mM D‐glucose) or in Barth's solution (88 mM NaCl, 1 mM KCl, 2.4 mM NaHCO_3_, 10 mM HEPES, 0.82 mM MgSO_4_, 0.33 mM Ca(NO_3_)_2_, 0.41 mM CaCl_2_) for experiments in oocytes to yield a 10 nM solution, which was immediately used.

### 2.3 Mouse hippocampal slice electrophysiology

After weaning, 3–10 weeks‐old mice (*n* = 21) were anesthetized with isoflurane, sacrificed, and the brains were quickly removed and subsequently processed in ice‐cold aCSF. The cerebellum was removed, and 300‐µm horizontal slices were cut with a Leica 1200S VT vibratome, typically between 900 and 1800 µm from the ventral part of the brain.

**FIGURE 1:**
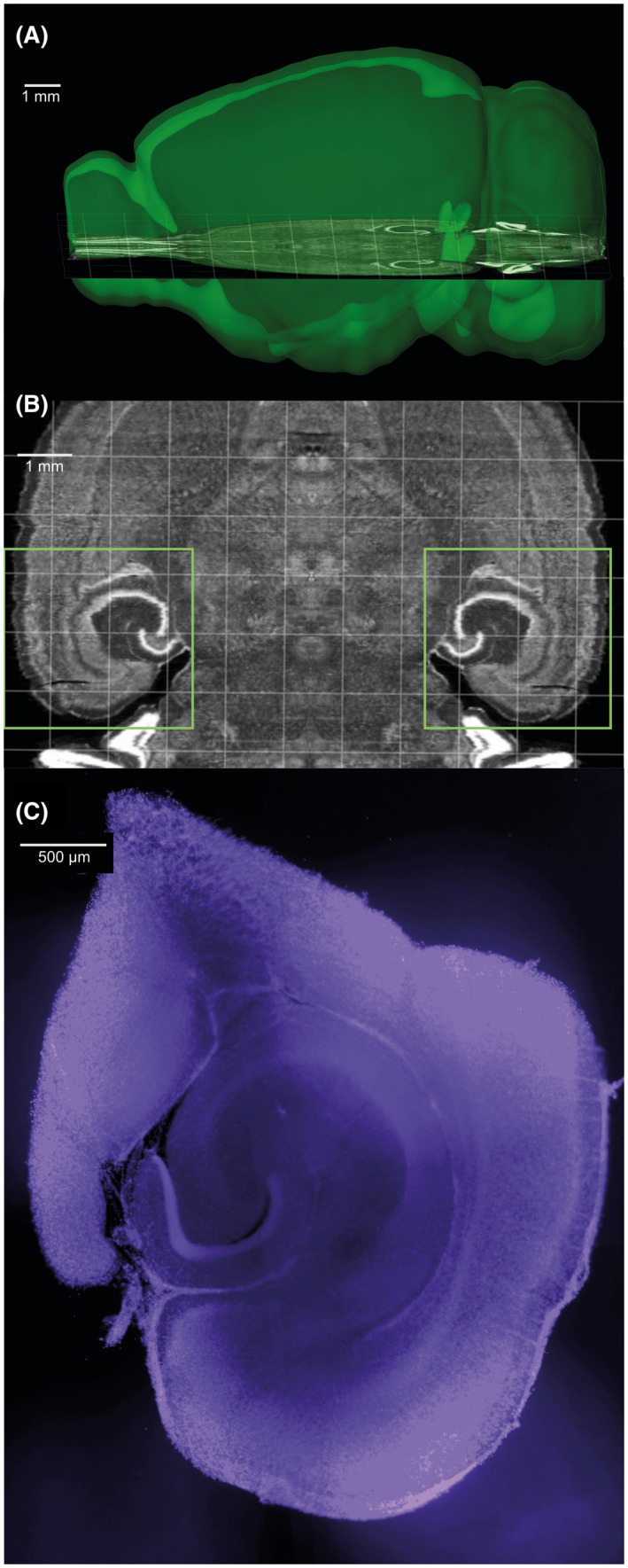
Images illustrating hippocampal slicing. (A and B) Approximate slicing location, according to the Allen Mouse Brain Atlas. Squares in B indicate the area of the slices kept. Images generated with the Allen Institute Brain Explorer 2 software, using the Allen Mouse Brain Atlas. (C) Example of slice section after electrophysiology (DAPI staining in blue)

Two to three slices containing the ventral hippocampus were prepared for electrophysiology. The slices (containing the posterior part of both hemispheres) were further dissected into two smaller slices of the hippocampus and peri‐hippocampal areas, with a sagittal‐plane cut medially to the outer shell of the dentate gyrus (Figure 1). This was done to enable easier transport between chambers, reduce the glucose and oxygen demands, as well as metabolite accumulation during incubation in the limited volume chamber.

From each animal, one or two slices (per condition) were incubated at room temperature (20° C) for 1 h in a small volume interface‐type chamber with 225 µl of aCSF, either with 10 nM PD1_n‐3DPA_ or vehicle (aCSF). The G_i_ protein inhibitor pertussis toxin (PTX, Tocris), 1 µg/ml was added together with PD1_n‐3DPA_ in a subset of slices. Preincubation of slices was preferred in this case, as opposed to perfusion with drug‐containing solutions in the recording chamber, because of the limited availability of the PD1_n‐3DPA_ compound, which also prompted the necessity of small incubation volumes.

### 2.4 Hippocampal slice electrophysiology

After incubation, slices were transferred to a submerged recording chamber constantly perfused with oxygenated aCSF and maintained at 34°C. Glass pipettes were pulled from thick‐wall Stoelting (ID/OD 0.75/1.50) or King Precision borosilicate glass (ID/OD 0.86/1.50) capillaries on a Sutter P‐97 puller and filled with a pipette solution containing CsCl 135 mM, NaCl 8 mM, CsOH EGTA 0.2 mM, CsOH HEPES 10 mM, MgATP 2 mM, Na_3_GTP 0.3 mM, QX‐314 5 mM, and 0.2% biocytin. Pipette resistance was within 2–5 MOhm.

Spontaneous and miniature post‐synaptic currents (PSCs) were recorded at 10 kHz, after a 3 kHz antialiasing Bessel filter, in gap‐free mode, from mid‐distal CA1 pyramidal neurons, voltage‐clamped at −70 mV, with either NBQX 5 µM/AP5 50 µM or PTX 100 µM in the perfusion medium. A HEKA EPC9 and HEKA Patchmaster v13.52 for Apple macOS was used for acquisition.

Post‐synaptic currents were detected with the correlation coefficient method^13^ and analyzed using Intel Python 3.6 under Windows 10 (code available, see^14^). Events were selected if their correlation coefficient with a double exponential fit of averaged post‐synaptic currents exceeded 0.6, with an amplitude greater than 3 pA (amplifier noise floor), 20%–80% rise time less than 5 ms, and halfwidth greater than the rise time, to exclude potential artifacts.

After filtering, the following numbers of events were analyzed. sIPSCs (spontaneous inhibitory post‐synaptic currents): 8 ctrl and 9 PD1_n‐3DPA_ incubated cells with 545 events each, mIPSCs (miniature inhibitory post‐synaptic currents): 5 ctrl and 8 PD1_n‐3DPA_ incubated cells with 929 events each, sEPSCs (spontaneous excitatory post‐synaptic currents): 9 ctrl and 9 PD1_n‐3DPA_ incubated cells with 646 events each, mEPSCs (miniature excitatory post‐synaptic currents): 8 ctrl and 8 PD1n‐3DPA incubated cells with 357 events each. For the rise time classification of fast and slow events, to ensure that we applied a threshold where ratio changes were approximately equal between groups and locally stable, thus not artificially inflating one of the categories, we applied the following steps:

For all possible cut‐off rise‐time values, τ_c_ in δτ intervals, where δ denotes the minimum discrete difference,

$$\tau_{c}=k∙\delta \tau \;,\;\;k\in N\;,k>0\;,\;\delta \tau >0$$

we calculate the ratio, $r_{s:f}$, of counts of observed rise‐times slower‐than to faster‐than τ_c_,

$$r_{s:f}=\frac{n_{\tau >\tau_{c}}+1}{n_{\tau \leq \tau_{c}}+1}$$

and select the minimum τ_c_ where the rates of change of $r_{s:f}$ for the 2 groups are equal or approximately equal, after the r_s:f_ spike at the beginning of the τ_c_ range:

$$\hat{\tau_{c}}=\min\left(\tau_{c}\right)\;:\;r_{s:f}\ll \max\left(r_{s:f}\right)\;,\;\frac{\delta r_{s:f,\;ctrl}}{\delta \tau }≅\frac{\delta r_{s:f,\;PD1}}{\delta \tau }$$

*δτ* = 100 µs (sampling rate limit).

The derivatives were plotted in logarithmic scale as $\frac{\delta \log_{10}\left(r_{s:f}\right)}{\log10\;∙\;r_{s:f}\;∙\;\delta \tau }$, to facilitate peak identification (not shown).

### 2.5 Xenopus oocyte electrophysiology

The solutions used for the oocyte electrophysiology experiments were prepared as follows. Oocyte ringer (OR) solution contained (in mM): NaCl 82.5; KCl 2.5; CaCl_2_ 2.5; MgCl_2_ 1; HEPES 5, adjusted to pH 7.4 with NaOH. Barth's solution contained (in mM): NaCl, 88, KCl, 1, NaHCO_3_, 2.4, HEPES, 10, MgSO_4_, 0.82, Ca(NO_3_)_2_, 0.33, CaCl_2_ 0.41.

GABA, AMPA, and cyclothiazide (CTZ) were purchased from Tocris (Bristol, UK). Salts for experimental solutions were purchased from Sigma–Aldrich (Merck Life Sciences, Milano, Italy).

Human hippocampal membranes were extracted from 3 patients who underwent surgery for drug‐resistant temporal lobe epilepsy (TLE). Human tissue was obtained as previously published^9^ and used in accordance with the Declaration of Helsinki. The Ethics Committee of the University of Rome “Sapienza” approved the technical procedures. Informed consent was obtained from all individuals involved in this study.

The preparation of membranes and the injection procedures into *Xenopus* oocytes have been previously described.^15^, ^16^ *Xenopus* oocytes were harvested and prepared as described previously.^9^, ^15^ In the experiments, a total of seven *Xenopus laevis* frogs have been used. Animal protocols were approved by the Italian Ministry of Health (authorization no. 427/2020‐PR). Twelve to 48 h after membrane injection, the oocytes were placed in a recording chamber (volume, 0.1 ml) and perfused continuously, 9–10 ml/min, with OR at room temperature (20–22 °C). The membrane currents were recorded from voltage‐clamped oocytes using two microelectrodes filled with 3 M KCl.^15^ GABA was applied at a final concentration of 500 µM for 4 s, AMPA was applied at 25 µM for 10 s, while oocytes were pretreated for 20 s with CTZ (25 µM). Neurotransmitters were freshly dissolved for each experiment.

PD1_n‐3DPA_ was applied to the incubation chamber for 5 min to 3 h or PD1_n‐3DPA_ was directly pressure‐injected into the oocytes (intracellular injection in filtered glycine 5 mM) with a glass micro‐pipette.^16^

### 2.6 GABA_A_ receptor Immunohistochemistry

Four male C57BL/6 mice, 11‐ to 16‐week‐old were used. Mice were decapitated and their brain removed from the skull. The brain was immediately immersed into ice‐cold oxygenated aCSF (95% O_2_/5% CO_2_), pH = 7.2 (*Tocris* Bioscience, Wiesbaden, Germany; 127 mM NaCl, 1.0 mM KCl, 1.2 mM KH_2_PO_4_, 26 mM NaHCO_3_, 2.4 mM CaCl_2_, 1.3 mM MgCl_2_). Brains were cut upside down in a Vibroslicer (Leica Microsystems VT1000S). From each mouse, five to six horizontal 400 µm sections of the ventral hippocampus were obtained. The sections were separated by hemisphere and about six sections from the left and six sections from the right hemisphere for each mouse brain were placed into individual wells. Three representative sections of the ventral hippocampus were selected from each hemisphere.

The slices were transferred to a 12‐well plate, each well containing 225 µl freshly prepared PD1_n‐3DPA_ solution or vehicle in oxygenated aCSF (3 sections/condition). The sections were incubated at room temperature for 60 min, then transferred to 12‐well plates containing PBS for 15 min, and to 4% paraformaldehyde (PFA) for 16 h. Finally, the sections were rinsed twice with PBS for 15 min and subsequently immersed into 20% sucrose for two subsequent 30 min epochs. Sections were kept in sucrose at 4–6°C for 3 days, then cut in a cryostat.

From each of 400 µm‐vibratome sections, three to five anatomically intact 40 µm microtome slices were kept in PBS containing 0.01% sodium azide at 4°C and used for immunofluorescence.

### 2.7 Immunofluorescence analysis

Immunofluorescence was performed as described previously.^17^ In brief, two slices from each hippocampal section were incubated free‐floating with 10% normal goat serum to reduce background (GIBCO #16210‐072, obtained through Fisher Scientific, Vienna, Austria) in Tris‐HCl‐buffered saline (TBS; 50 mm), pH 7.2, containing 0.4% Triton X‐100 (TBS‐Triton) for 90 min, followed by incubation with respective to a rabbit antibody raised against a 7His‐fusion protein of amino acids 316–352 of the γ2 subunit of the rat GABA receptor protein with maltose‐binding protein (final concentration 1 µg/ml^18^ at room temperature for 16 h, followed by washing with TBS‐Triton).^19^ Then, slices were incubated with HRP‐coupled donkey anti‐rabbit secondary antibody (1:500; catalog #711035152, Jackson ImmunoResearch; RRID:AB_10015282) at room temperature for 120 min and then reacted with TSA‐Cy3 (homemade; Lumiprobe; 1:100 in 50 mm PBS and 0.005% H_2_O_2_) at room temperature for 5 min. Sections were mounted on slides and covered using Vectashield mounting medium (Vector Laboratories, Inc., Burlingame, USA).

### 2.8 Determination of relative optic densities (ROD)

The sections were analyzed using a fluorescence microscope. We took black and white images of the hippocampus at 25X magnification revealing images with bright immunoreactive areas and dark backgrounds. The images were evaluated using the Fiji/ImageJ program.^20^ We determined gray values within strata oriens and radiatum CA1 and CA3, the stratum lacunosum‐moleculare CA1, and the dentate molecular layer within representative areas (using a circular selection tool). As background levels, we defined gray values obtained in the hilus of the dentate gyrus of the same sections containing no immunoreactivity. After deducting these backgrounds, gray values were converted to relative optic densities (ROD) using the formula ROD = log_10_(256/(255‐grey value)).

### 2.9 Calculation of mean RODs

From each mouse, three 400 µm vibratome sections had been taken and immunoreactivity was determined in two 40 µm cryotome sections obtained from each vibratome section. First, ROD values obtained from the two 40 µm cryotome sections were averaged (each value representing one vibratome section). Then, the mean ROD values were calculated from the corresponding three vibratome sections (obtained from the same mouse). The resulting values represented the RODs of the individual mice. These were averaged (four PD1_n‐3DPA_ incubated vs. four aCSF incubated).

### 2.10 Statistics

Results are presented as mean ± standard error of the mean.

The electrophysiology data are depicted in bar‐whisker plots as the median, range, and individual data points of average values per cell, unless otherwise indicated. Mann‐Whitney U test was used to compare groups and the Kolmogorov‐Smirnov test for Empirical Cumulative Distribution comparisons. The differences were considered as statistically significant with a *p*‐value <.01, and *D*‐value >.05. Fisher's exact test was used for the analysis of proportion changes.

For the slice incubation and immunohistochemistry experiment, statistics were done by Student's t‐test. Differences between groups were considered significant for values of *p* < .05.

## 3 RESULTS

### 3.1 PD1_n‐3DPA_ enhances inhibitory synaptic inputs to CA1 pyramidal neurons

**FIGURE 2:**
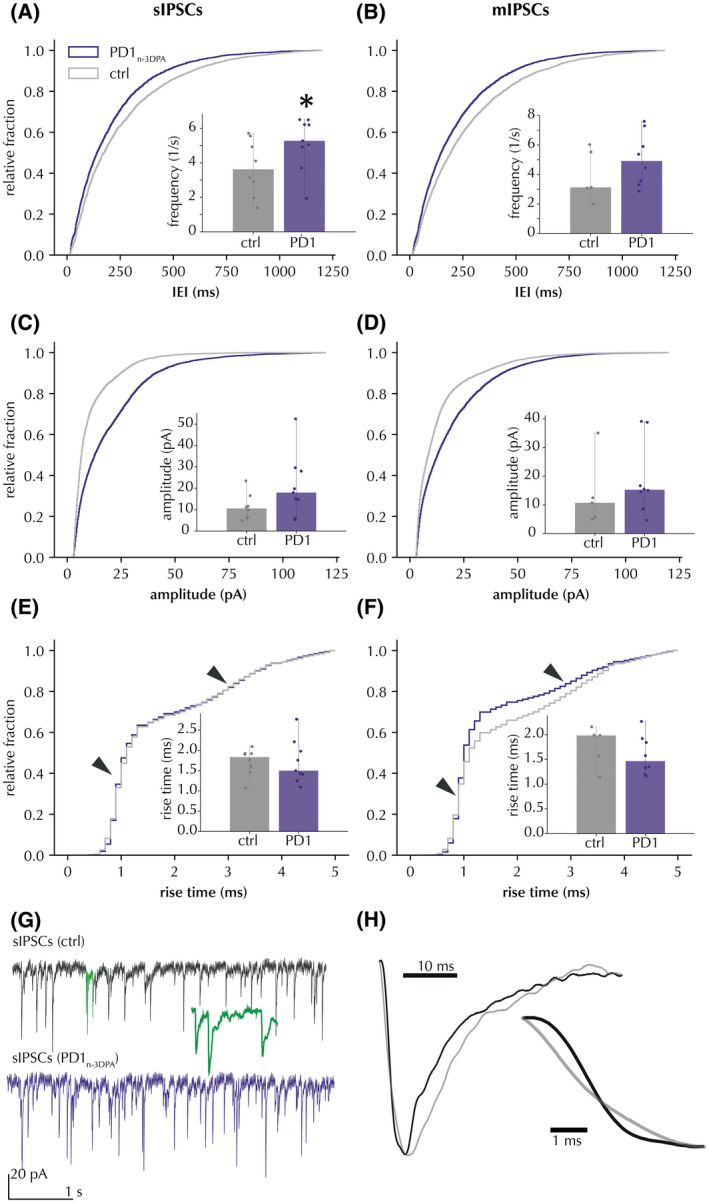
PD1_n‐3DPA_ incubation increases the frequency and amplitude of IPSCs recorded in CA1 pyramidal neurons. (A) sIPSC interevent interval (IEI) presented as cumulative probability curves (Kolmogorov‐Smirnov test *D* = .098, *p* < .01) and cell‐based averages (insert) of sIPSC frequency. (B) mIPSCs, same presentation as (A), IEI (Kolmogorov‐Smirnov test *D* = .102, *p* < .01) and average frequencies. (C and D) sIPSC and mIPSC amplitudes, respectively, presented as cumulative probability curves (Kolmogorov‐Smirnov test sIPSCS: *D* = .272, *p* < .01; mIPSCs: *D* = .181, *p* < .01) and cell‐based averages (insert). (E and F) Rise‐times of sIPSCs and mIPSCs, respectively, presented as cumulative probability curves (Kolmogorov‐Smirnov test sIPSCS: *D* = .029, *p* > .01; mIPSCs: *D* = .101, *p* < .01) and cell‐based averages (inserts). Arrows denote 2 distinct distribution peaks around 1 and 3 ms. Insets display the median and range of cell averages. * Mann‐Whitney *p* < .05 for cell averages. (G) representative traces of sIPSCs with and without PD1_n‐3DPA_. Highlighted segment (green) shows 10‐fold time‐stretched recording. (H) magnification of amplitude‐normalized averaged fast (black) and slow (grey) rise‐time events in cells from control slices (*n* = 125 sIPSCs per group)

Incubation with PD1_n‐3DPA_ increased inhibitory drive onto pyramidal neurons as revealed by decreased interevent interval (IEI) of sIPSCs in cumulative probability curves (Figure 2A) and increased cell‐based average frequency of sIPSCs (Figure 2A insert, Table 1). The mIPSCs, however, showed decreased IEI but no change in cell‐based average frequency (Figure 2B insert). The amplitude of sIPSCs and mIPSCs showed an increase after PD1_n‐3DPA_ exposure only when analyzed by cumulative probability distributions but failed to demonstrate significant differences when cell‐based mean values were compared (Figure 2C,D inserts). Representative traces of spontaneous (s)‐ and miniature (m)IPSCs are presented in Figure 2G. These data indicate that the most pronounced changes occurred in sIPSC frequencies, as confirmed by both distribution and cell‐average analysis, while other changes were only apparent by distribution analysis and not by cell‐based averages. Thus, we conclude that PD1_n‐3DPA_ exposure affected predominantly the frequency, and to a lesser extent the amplitude of the sIPSCs. Taken together, these data would support that most of the PD1_n‐3DPA_‐induced changes occurred in the presynaptic site of inhibitory synapses.^21^

**TABLE 1:** Frequency and amplitude comparison of cell‐averaged IPSCs between control and PD1_n‐3DPA_‐treated slices

	Frequency (1/s)		Amplitude (pA)	
	sIPSCs	mIPSCs	sIPSCs	mIPSCs
Vehicle	3.717 ± 0.536, *n* = 8 (7)	3.949 ± 0.695, *n* = 5 (5)	11.266 ± 2.048, *n* = 8 (7)	13.804 ± 4.926, *n* = 5 (5)
PD1_n‐3DPA_	5.143 ± 0.476, *n* = 9 (6)	5.048 ± 0.595, *n* = 8 (5)	20.98 ± 4.541, *n* = 9 (6)	19.133 ± 4.263, *n* = 8 (5)
Mann–Whitney U test p	0.046	0.171	0.068	0.136

Note

Numbers of animals in parentheses.

**FIGURE 3:**
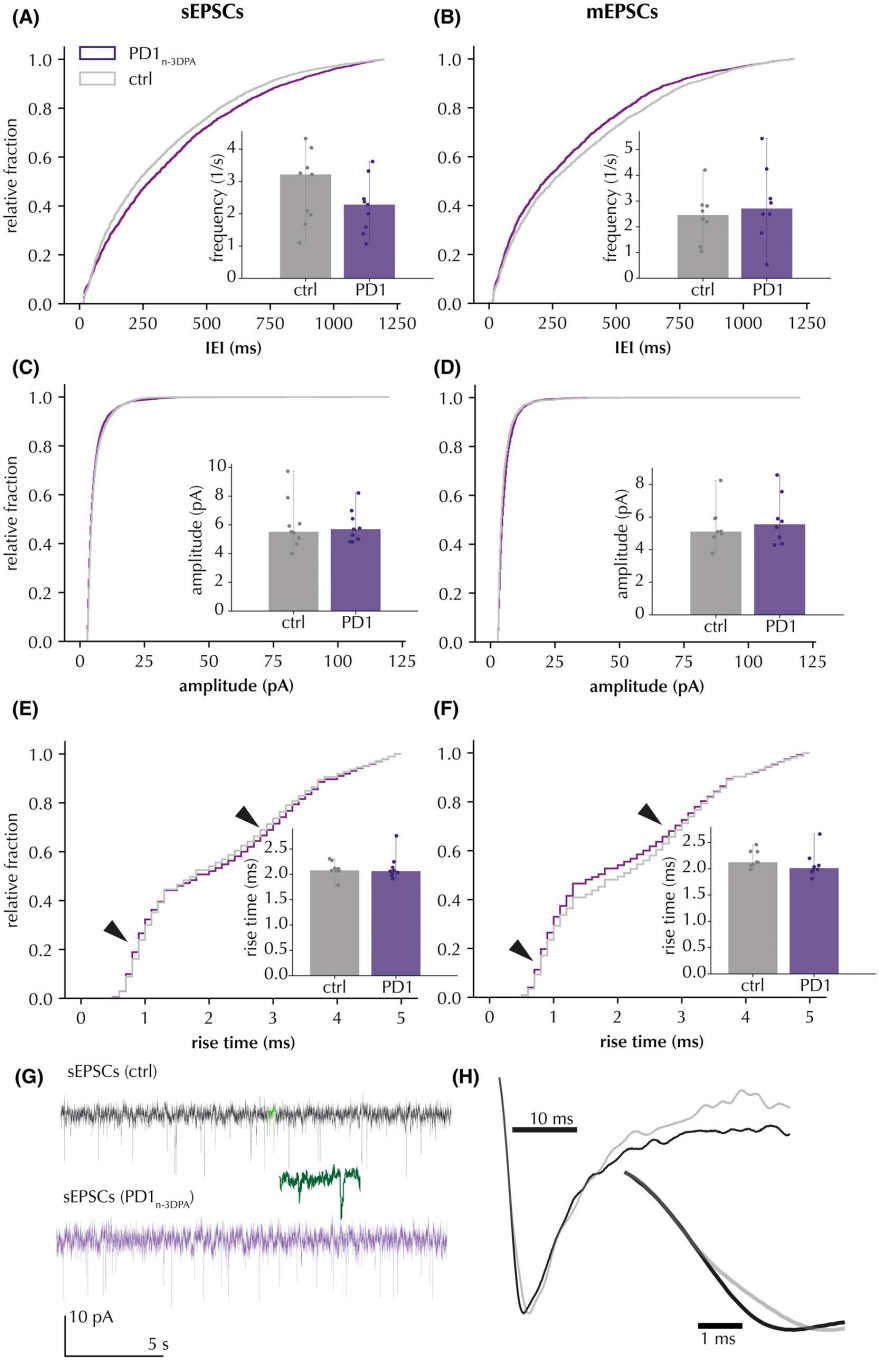
Effect of PD1_n‐3DPA_ incubation on EPSCs recorded in CA1 pyramidal neurons. (A and B) sEPSCs and mEPSCs, respectively, are presented as cumulative probability curves for IEI (sEPSCs: Kolmogorov‐Smirnov test *D* = .014, *p* < .01; mEPSCs: *D* = .045, *p* < .01) and cell‐based averages for sEPSC frequencies (inserts). (C and D) sEPSCs and mEPSCs, respectively, are presented as cumulative probability curves (sEPSCs: Kolmogorov‐Smirnov test *D* = .045, *p* < .01; mEPSCs: *D* = .089, *p* < .01) for amplitudes and cell‐based averages (inserts). (E and F) sEPSC and mEPSC rise‐times, respectively, are presented as cumulative probability curves (sEPSCs: Kolmogorov‐Smirnov test *D* = .028, *p* > .01; mEPSCs: *D* = .062, *p* < .01) and cell‐based averages. Insets display the median and range of cell average. Arrows denote much less pronounced peaks as compared to those in Figure 1. (G) representative traces with and without PD1_n‐3DPA_. Highlighted segment (green) shows 10‐fold time‐stretched recording. (H) magnification of amplitude‐normalized averaged fast (black) and slow (grey) rise‐time events in cells from control slices (*n* = 42 sEPSCs per group)

A similar analysis for IPSCs was also applied to excitatory post‐synaptic currents (EPSCs) (Figure 3). The results showed no statistically significant changes neither for cumulative probability curves (both for sEPSCs, nor for mEPSCs, respectively), nor for cell‐based averaged comparisons of IEI (Figure 3A,B). Similarly, there was no significant effect on sEPSC amplitudes (Figure 3C). There was a minor increase in mEPSC amplitudes according to the Kolmogorov‐Smirnov test (with *D* = .089) which was not confirmed by cell‐based average analysis (Figure 3D). Also, sEPSC kinetics were unchanged (Figure 3E), while some minor changes in distributions were detected for mEPSCs in the Kolmogorov‐Smirnov test (*D* = .062). Overall, incubation of hippocampal slices with PD1_n‐3DPA_ had no effect on sEPSCs and minor, if any effect, on mEPSCs. These minor changes in mEPSCs were negligible as compared to those for IPSCs (see Table 2).

**TABLE 2:** Frequency and amplitude comparison of cell‐averaged EPSCs between control and PD1_n‐3DPA‐_treated slices

	Frequency (1/s)		Amplitude (pA)	
	sEPSCs	mEPSCs	sEPSCs	mEPSCs
Vehicle	2.789 ± 0.351, *n* = 9 (6)	2.403 ± 0.329, *n* = 8 (5)	6.038 ± 0.553, *n* = 9 (6)	5.48 ± 0.433, *n* = 8 (5)
PD1_n‐3DPA_	2.232 ± 0.266, *n* = 9 (5)	2.870 ± 0.491, *n* = 8 (4)	5.897 ± 0.358, *n* = 9 (5)	5.822 ± 0.507, *n* = 8 (4)
Mann Whitney *U* test *p*	.189	.215	.500	.479

Note

Numbers of animals in parentheses.

To address the question of whether the PD1_n‐3DPA_‐induced changes in inhibitory transmission were uniformly distributed among peri‐somatic and dendritic inhibitory synapses on the pyramidal neurons, we analyzed IPSC kinetics. The cumulative probability curves of IPSC rise‐times revealed two inflections of the curves (Figure 2E, arrows) around 1 and 3 ms. This suggests that there were two populations of IPSCs with different rise‐time kinetics: “fast” and “slow,” respectively (Figure 2H). These IPSCs were interpreted as representing peri‐somatic (fast) or dendritic (slow) synapse activity, the latter being a result of passive filtering because of more remote location from the recording patch pipette attached to the cell soma. Interestingly, we observed an increase in rising times for mIPSCs after PD1_n‐3DPA_ incubation (Figure 2F), suggesting that PD1_n‐3DPA_ might preferentially affect peri‐somatic synapses.

**FIGURE 4:**
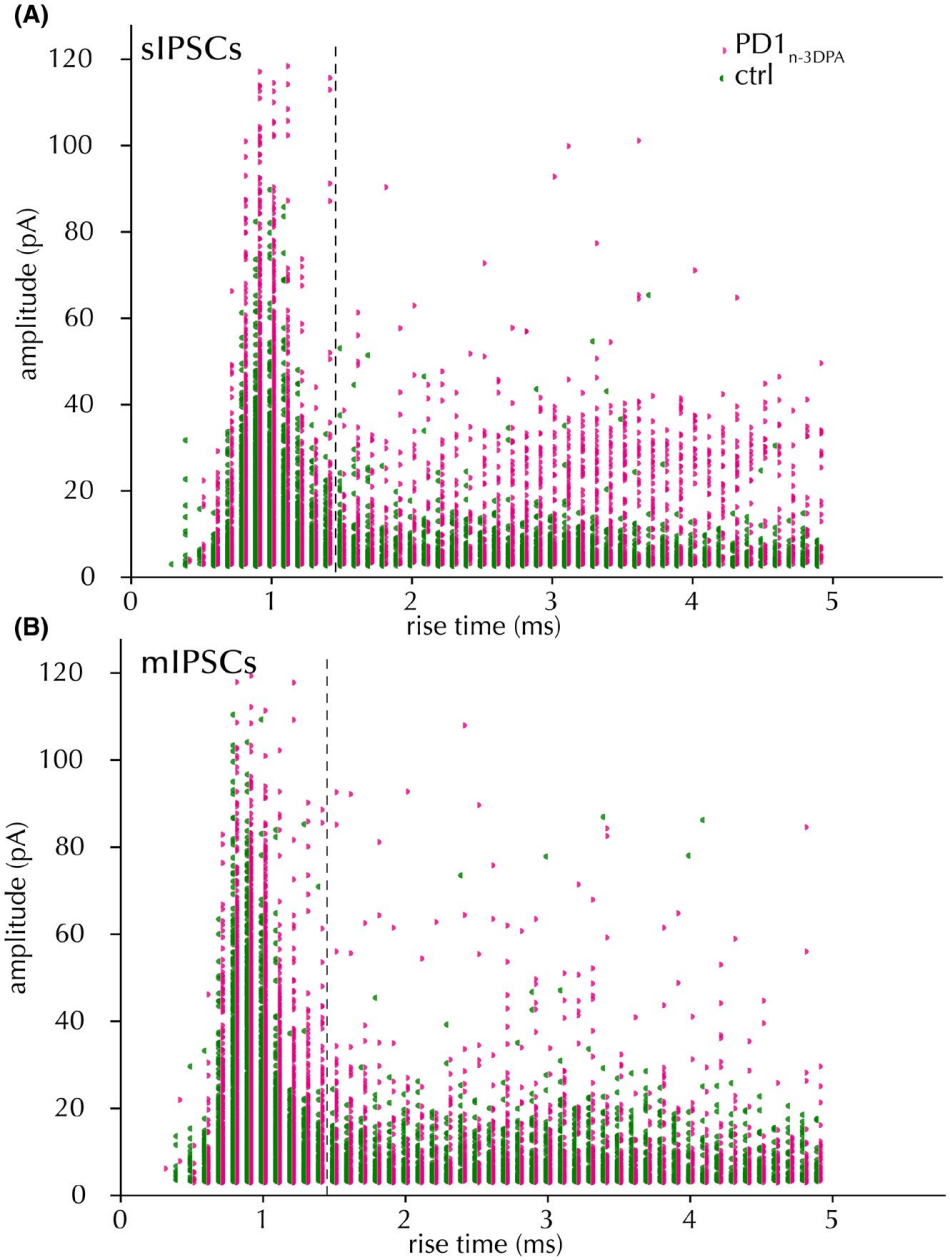
Rise time/amplitude distribution of IPSCs. Spontaneous (A) and miniature (B) IPSCs, after vehicle (green) and PD1_n‐3DPA_ incubation (magenta). Fisher's exact test ns (sIPSCs), *p* < .05 (mIPSCs)

In principle, perisomatic IPSCs with fast kinetics should also display larger amplitudes. Indeed, when we plotted IPSC amplitudes against rising times, the high amplitude and fast rise time events had higher amplitude than those with slow kinetics (Figure 4A,B). Notably, IPSCs with faster kinetics and higher amplitudes displayed a larger increase in amplitudes after PD1_n‐3DPA_ exposure compared to those with slow kinetics and low amplitudes (Figure 4A,B, magenta). However, only the increase in mIPSCs was statistically significant (see Table 3 for details). This would support that preferentially perisomatic inhibitory synapses onto the CA1 pyramidal neurons were affected by PD1_n‐3DPA_ incubation.

**TABLE 3:** Increased proportion of fast rise‐time IPSC counts following PD1_n‐3DPA_ incubation

	sIPSCs		mIPSCs	
	Fast	Slow	Fast	Slow
Vehicle	2734	1619	2777	1864
PD1_n‐3DPA_	3110	1787	5190	2235
Fisher's exact *p*	.490		<.001	

Note

Rise times separated at 1.4 ms.

### 3.2 GABA_A_ receptor immunohistochemistry

**FIGURE 5:**
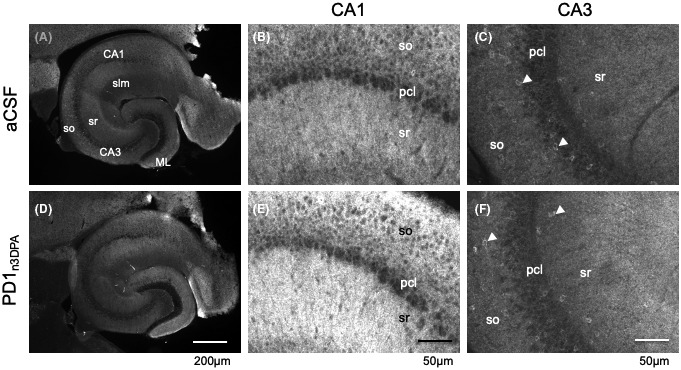
Examples of slices and the subfields CA1 and CA3 examined by immunofluorescence for GABA_A_ receptor γ2‐subunit. Panels A and C show immunofluorescence images of representative slices incubated with aCSF (A) or PD1_n3DPA_ in aCSF (D). Panels B and E show respective details of Sector CA1 and panels C and F of sector CA3 of the same sections. GABA_A_ receptor γ2‐immunofluorescence labels primarily dendrites (note punctate labeling in B and E). Pericarya of pyramidal cells and interneurons are mostly unlabeled and appear dark. Note that in CA3 some interneurons show immunoreactivity at their cell bodies (arrow heads). Panel E indicates somewhat more intensive labeling for the γ2‐subunit as observed in some sections. CA1, CA3, cornu ammonis, sectors 1 and 3; ML, dentate molecular layer; pcl, pyramidal cell layer; slm, stratum lacunosum moleculare; so, stratum oriens; sr, stratum radiatum

Although the results of electrophysiological experiments pointed towards a preferential presynaptic mode of PD1_n‐3DPA_ action, cumulative probability curves suggested a potential effect on IPSC amplitudes; therefore, postsynaptic changes (e.g., in GABA_A_ receptors) could also be present. Consequently, we investigated whether PD1_n‐3DPA_ could have induced changes in postsynaptic GABA_A_ receptor subunit composition. GABA_A_ receptors are the major inhibitory receptors on principal neurons and represent chloride channels assembled by five heterogeneous subunits. These subunits derive from about 15 different genes. A majority of GABA_A_ receptors contain a γ‐subunit.^18^ Together with a variety of scaffold proteins, like gephyrin, this subunit appears to contribute to the assembly of the receptor complex.^22^, ^23^ We, therefore, incubated hippocampal slices with PD1_n‐3DPA_ and by immunofluorescence assessed the expression of the GABA_A_ receptor γ‐subunit in six different subfields (Figure 5) of the hippocampus (Table 4).

**TABLE 4:** GABAA receptor γ2‐subunit immunoreactivity in slices of the ventral hippocampus after incubation with PD1n‐3DPA Data represent the mean ± SEM of values from four vibratome slices (incubated either with aCSF or PD1_n‐3DPA_) each corresponding to an individual mouse

	DG‐ML	CA1 so	CA1 sr	CA1 slm	CA3 so	CA3 sr
aCSF	0.200 ± 0.019	0.217 ± 0.038	0.251 ± 0.037	0.213 ± 0.024	0.164 ± 0.019	0.160 ± 0.020
PD1_n‐3DPA_	0.229 ± 0.011 (115%)	0.243 ± 0.037 (112%)	0.282 ± 0.007 (112%)	0.215 ± 0.023 (99%)	0.125 ± 0.007 (76%)	0.165 ± 0.020 (102%)
*p*‐value	.236	.531	.363	.973	.089	.861

Note

Each data point is based on average ROD values obtained from immunofluorescence from five to six 40‐µm sections/vibratome slice. Percentage values are relative to aCSF incubation.

Abbreviations: DG‐ML, molecular layer of the dentate gyrus; slm, stratum lacunosum moleculare; so, stratum oriens; sr, stratum radiatum.

Following PD1_n‐3DPA_ incubation, minor fluorescence differences were observed in all hippocampal fields, compared to control slices, and these alterations were not statistically significant (Table 4). The representative slices with immunofluorescence staining in grayscale are presented in Figure 5. The data, therefore, did not support major changes in GABA_A_ receptor γ‐subunit composition, indirectly reinforcing the preferentially presynaptic effect of PD1_n‐3DPA_.

### 3.3 Xenopus oocyte electrophysiology

To further strengthen the immunostaining results, we performed oocyte electrophysiology since the oocyte membranes incorporate exogenous neuronal membranes fragments containing GABA_A_ and glutamate receptors.^15^, ^24^ We examined GABA and AMPA currents in oocytes incubated with PD1_n‐3DPA_ or after intra‐oocyte injection of PD1_n‐3DPA_ to test if there was an intracellular site of PD1_n‐3DPA_ interaction with the membrane receptors. No significant changes either in GABA or AMPA currents were observed in these experiments at any timepoint of incubation (Table 5). These data support the slice electrophysiology results that PD1_n‐3DPA_‐induced changes in IPSCs are generated at presynaptic sites and that PD1_n‐3DPA_ does not alter postsynaptic GABA_A_ receptors. Moreover, PD1_n‐3DPA_ appears ineffective on glutamatergic excitatory synaptic transmission.

**TABLE 5:** Results of PD1_n‐3DPA_ incubation and its intracellular injection in oocytes injected with human hippocampal tissue

Incubation time	GABA current (pre‐PD1_n‐3DPA_)	GABA current (post‐PD1_n‐3DPA_)	AMPA current (pre‐PD1_n‐3DPA_)	AMPA current (post‐PD1_n‐3DPA_)
5 min	87.7 ± 16.0 nA (*n* = 6)	88.3 ± 14.0 nA (*n* = 6)	–	–
20 min	54.9 ± 23.1 nA (*n* = 6)	54.4 ± 18.9 nA (*n* = 6)	–	–
1 h	75.0 ± 10 nA (*n* = 7)	77.0 ± 8 nA (*n* = 7)	19.3 ± 1.5 nA (*n* = 5)	18.3 ± 1.8 (*n* = 5)
2 h	54.7 ± 15.7 nA (*n* = 18)	54.3 ± 13.9 nA (*n* = 18)	26.9 ± 3.8 nA (*n* = 6)	23.0 ± 5,.2 nA (*n* = 6)
3 h	58.5 ± 17.0 nA (*n* = 17)	57.0 ± 15.5 nA (*n* = 17)	29.4 ± 3.5 nA (*n* = 6)	23.0 ± 7.5 nA (*n* = 6)
Intracellular injection	70.7 ± 20.4 nA (*n* = 7)	64.8 ± 22.7 nA (*n* = 7)	7.6 ± 1.7 nA (*n* = 4)	6.2 ± 1.5 nA (*n* = 4)

### 3.4 PD1_n‐3DPA_ effect is blocked by Pertussis toxin

**FIGURE 6:**
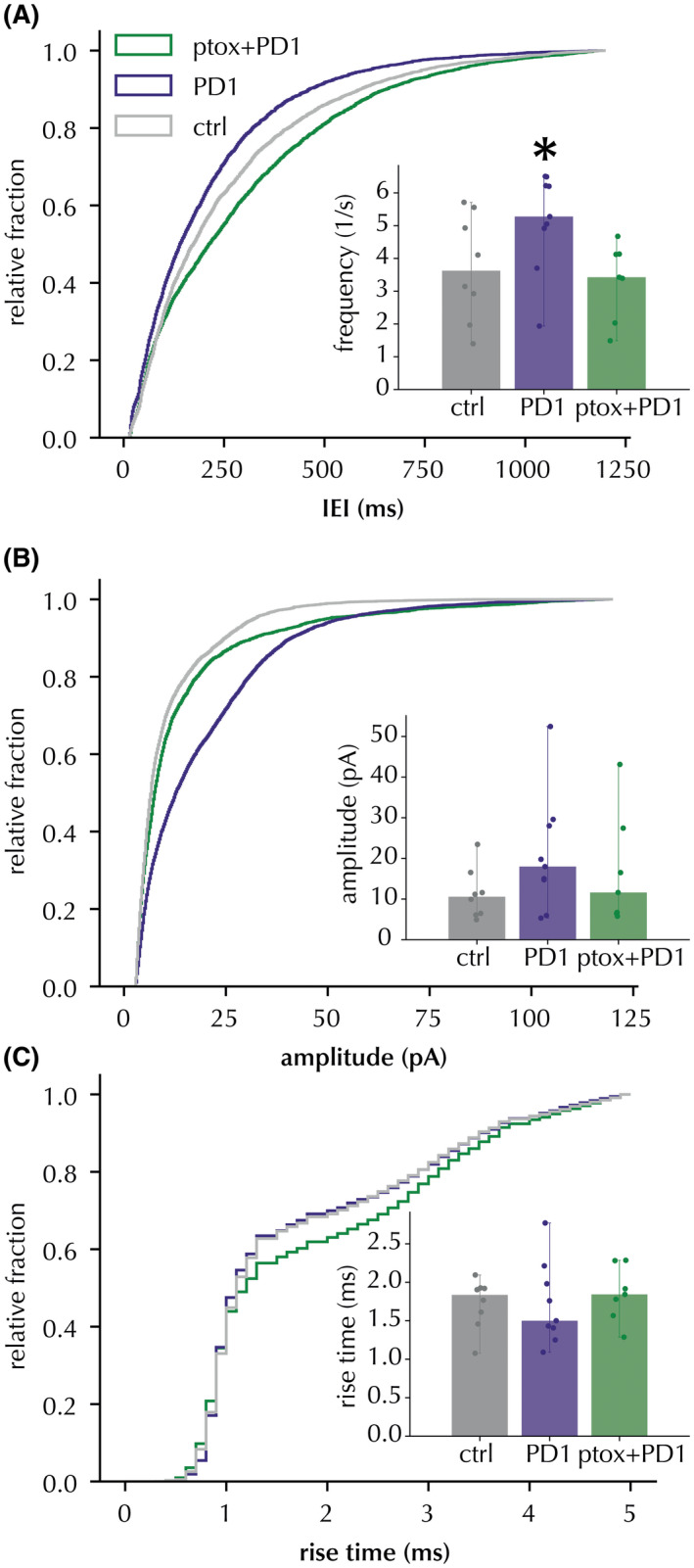
PD1_n‐3DPA_ effect on IEI of IPSC in CA1 pyramidal neurons was reverted by pertussis toxin. Cumulative distribution curves of IEI (A), amplitudes (B) and rise times (C) of IPSCs were recorded after incubation with PD1_n‐3DPA_ or with PD1_n‐3DPA_ + PTX. The control (vehicle) group is displayed as a reference. Insets display the median and range of cell averages. * Mann‐Whitney *p* < .05 for cell averages (PD1_n‐3DPA_ vs. PD1_n‐3DPA_ + PTX), Kolmogorov‐Smirnov *p* < .01, *D* > .1 for all PD1_n‐3DPA_ vs. PD1_n‐3DPA_ + PTX distribution comparisons

Next, we hypothesized that the PD1_n‐3DPA_ effect could be mediated via some type of GPCRs. Since, to our best knowledge, there are no data available on which type of GPCR could be involved in PD1_n‐3DPA_ signaling, we used pertussis toxin, a G_i_ inhibitor, to address this question. Pertussis toxin (1 µg/ml) incubation reversed the increase in frequency (both cumulative probability curves and cell‐based average frequency), amplitude, and kinetics (only cumulative probability curves) of spontaneous IPSC induced by PD1_n‐3DPA_ (Figure 6) (K‐S *p* < .001 for distribution changes relative to PD1_n‐3DPA_ incubation, cell‐averaged comparison statistically significant for the frequency reversal–Table 6). These data suggest that PD1_n‐3DPA_ increases inhibitory drive onto the pyramidal neurons by interaction with a GPCR. Further studies are required, however, to identify the specific type of the GPCR that is responsible for this effect.

**TABLE 6:** Cell‐averaged sIPSC frequency and amplitude comparison between PD1_n‐3DPA_ and PD1_n‐3DPA_ + pertussis toxin incubations. *n* = 545 events per cell

	Frequency (1/s)	Amplitude (pA)
PD1_n‐3DPA_	5.143 ± 0.476, *n* = 9 (6)	20.98 ± 4.541, *n* = 9 (6)
PD1_n‐3DPA_ + pertussis toxin	3.324 ± 0.408, *n* = 7 (5)	16.797 ± 4.874, *n* = 7 (5)
Mann Whitney U test p	0.010	0.263

Note

Numbers of animals in parentheses.

## 4 DISCUSSION

Here, we demonstrate that PD1_n‐3DPA_ applied to hippocampal slices enhances inhibitory drive onto the CA1 pyramidal neurons. Interestingly, most of this increase was observed in peri‐somatic inhibitory synapses. No changes were observed in excitatory synapses onto the pyramidal neurons. In addition, we demonstrate that this effect of PD1_n‐3DPA_ is mediated by a yet unidentified GPCR.

Our findings provide fresh evidence that, in addition to its previously reported pro‐resolving and anti‐inflammatory actions, PD1_n‐3DPA_ mediates a direct effect on network excitability, by interacting with GPCR. This newly described mechanism of action of PD1_n‐3DPA_ may contribute to the anti‐seizure and neuroprotective effects described in vivo.^6^ Notably, PD1_n‐3DPA_ was among the most abundant lipid mediators measured during epilepsy development in mice suggesting it might be a key player in the tissue attempt to engage resolution pathways. Accordingly, early post‐injury administration of PD1_n‐3DPA_ significantly reduced neuroinflammation, in line with previous findings that PD1_n‐3 DPA_ and its precursor n‐3 DPA^25^ exert powerful anti‐inflammatory effects.^26^ This anti‐inflammatory effect was associated with a drastic reduction in the number and duration of spontaneous seizures in mice. The 10‐nM concentration we used in hippocampal slices was previously shown to mediate the anti‐inflammatory effects of the molecule in vitro.^27^ When PD1_n‐3DPA_ was applied in vivo (200 ng, 552.5 µM injected concentration)^6^ into the ventricle, the original concentration was diluted in the CSF, which in mice is approximately 35 µl,^28^ resulting in a final concentration of ~15.8 µM. However, PD1_n‐3DPA_ had to diffuse to the hippocampal tissue, along a concentration gradient defined by the distance to the target tissue, diffusion coefficient, etc. resulting in further dilution. Therefore, it is difficult to compare the in vivo concentration effective on seizures with the in vitro concentration affecting GABA neurotransmission. However, the concentration we applied to the slices has been defined as optimal by the previous in vitro reports^27^ and appears to be sufficient to elicit an effect. Therefore, the mechanism of action of PD1_n‐3DPA_ identified by the present in vitro study is likely to be relevant in vivo when PD1_n‐3DPA_ is injected into the ventricles and suppresses seizures. Thus, increased inhibitory drive on pyramidal neurons of the hippocampus caused by direct application of PD1_n‐3DPA_ may counteract increased network excitability and thereby reduce spontaneous recurrent seizures.

An interesting finding of this study is that the effect of PD1_n‐3DPA_ was predominantly exerted on perisomatic inhibitory synapses. Although our data on cumulative probability curves demonstrating increased amplitude of IPSCs may also indicate a postsynaptic action of PD1_n‐3DPA_ on inhibitory synapses, both immunohistochemical analyses of postsynaptic GABA_A_ receptors and Xenopus oocyte assay failed to demonstrate changes at postsynaptic sites. The oocyte assay has been proven to be effective in identifying alteration in GABA_A_ receptor function.^24^ These data exclude the possibility of a direct action of the compound on these receptor populations, however indirect metabotropic (i.e., on GABA_B_ receptor expression) or direct intracellular effects cannot be excluded. One could speculate that PD1_n‐3DPA_ acts therefore mostly presynaptically on CA1 pyramidal neurons, and selectively affects those inhibitory interneurons that innervate the perisomatic area of the principal neurons, such as, for example, parvalbumin‐ or cholecystokinin‐expressing basket cells.^29^, ^30^ Since we show that the PD1_n‐3DPA_ effect is sensitive to pertussis toxin, it implies that these interneurons may selectively express a specific type of GPCRs and/or associated G_o_/G_i_ proteins that mediate the PD1_n‐3DPA_ effect. This hypothesis is worth further investigation.

In view of its neuromodulatory effects, PD1_n‐3DPA_ may prove to be an attractive target for anti‐ictogenic and anti‐epileptileptogenic interventions. In this regard, identifying a specific GCPR that mediates its effect would be of particular interest.

Our data raise the possibility that PD1_n‐3DPA_ might also counteract in vivo the effects of ictogenic cytokines such as IL‐1β and TNF which are proven to decrease inhibitory synaptic transmission.^5^

In conclusion, we have identified a novel mechanism by which a key pro‐resolving molecule affects network excitability, in addition to its anti‐inflammatory effects. This direct neuronal mechanism involves yet unidentified a GPCR, elucidation of which may provide a novel target for anti‐epileptogenic and anti‐ictogenic approaches in epilepsy.

## DISCLOSURES

The authors have no relevant financial or non‐financial interests to disclose.

## AUTHOR CONTRIBUTIONS

Designed research: Merab Kokaia, Annamaria Vezzani, and Jesmond Dalli; performed research: Apostolos Mikroulis, Marco Ledri, Gabriele Ruffolo, Eleonora Palma, and Günther Sperk; analyzed data: Apostolos Mikroulis, Gabriele Ruffolo, Eleonora Palma, and Günther Sperk; wrote the paper: Apostolos Mikroulis, Marco Ledri, Gabriele Ruffolo, Eleonora Palma, Günther Sperk, Jesmond Dalli, Annamaria Vezzani, and Merab Kokaia.

## Data Availability

The data that support the findings of this study are available on request from the corresponding author. Code is available at GitHub and Zenodo with DOI: 10.5281/zenodo.4431620.
